# Preparation of bioplastic consisting of salmon milt DNA

**DOI:** 10.1038/s41598-022-11482-4

**Published:** 2022-05-06

**Authors:** Masanori Yamada, Midori Kawamura, Tetsuya Yamada

**Affiliations:** 1 Department of Chemistry, Faculty of Science, Okayama University of Science, Ridaicho, Kita-ku, Okayama, 700-0005 Japan; 2 Research Faculty of Agriculture, Hokkaido University, Sapporo, 060-8589 Japan

**Keywords:** Environmental sciences, Chemistry, Materials science

## Abstract

The microplastic that pollutes the ocean is a serious problem around the world. The bioplastic consisting of biopolymers which is degraded in nature, is one of the strategies to solve this problem. Although the bioplastics consisting of protein, polysaccharide, polylactic acid, etc*.*, have been reported, which consist of DNA, one of the most important materials in the genetic process, have not been reported to the best of our knowledge. In addition, a large amount of DNA-containing materials, such as salmon milts, is discarded as industrial waste around the world. Therefore, we demonstrated the preparation of a bioplastic consisting of salmon milt DNA. The DNA plastic was prepared by the immersion of a DNA pellet in a formaldehyde (HCHO) solution and heating. As a result, the water-stable DNA plastics were obtained at the HCHO concentration of 20% or more. Particularly, the DNA plastic with a 25% HCHO treatment showed water-insoluble, thermally stable, and highly mechanical properties. These are due to the formation of a three-dimensional network via the crosslinking reaction between the DNA chains. In addition, since DNA in plastic possesses the double-stranded structure, these plastics effectively accumulated the DNA intercalator, such as ethidium bromide. Furthermore, the DNA plastics indicated a biodegradable property in a nuclease-containing aqueous solution and the biodegradable stability was able to be controlled by the HCHO concentration. Therefore, salmon milt DNA has shown the potential to be a biodegradable plastic.

## Introduction

DNA is one of the important materials related to the genetic process in living things^1^. Since the DNA possesses various specific functions, such as the formation of the double-stranded structure, the complementary interaction between nucleobases, the selective binding of metal ions, etc., it has the potential to be used as a functional material^2,3^. In addition, a large amount of DNA-containing materials, such as salmon milts and shellfish gonads, is discarded as industrial waste around the world. Therefore, the conversion of discarded DNA into a useful material has attracted attention as a novel material development^2,3^. These DNAs have been used as functional materials, such as the accumulations of planar structure-containing harmful compounds^4^, the selective removals of harmful metal ions^5^, ion conducting materials^6^, hydrogels^7^, liquid crystals^8,9^, electric devices^10^, optical materials^11^, bio- and medical materials^12,13^, etc. In addition, recently, DNA attracted attention as a nano-building block in the field of nanotechnology and various nano-materials consisting of DNA have been reported^14,15^. However, the utilization of DNA in an industrial product, such as plastic, has not been reported to the best of our knowledge.

Generally, artificial plastics are mainly obtained from petroleum. These plastics have many advantages, such as light weight, non-corrosive, low cost, high mechanical strength, easy processing, etc^16^. Therefore, a large amount of plastics has been produced as industrial products. However, artificial plastics possess various problems, such as the depletion of petroleum resources, non-degradation in nature, low chemical and thermal resistance, etc^17–19^. Particularly, the microplastics that pollute the ocean are a serious problem around the world^19,20^. These microplastics, which are non-biodegradable in nature, can enter the body via bioconcentration or bioaccumulation and are thought to have a negative impact on living organisms^19,20^. Although there are various causes for microplastics, one of which is related to their non-degradation in nature. Therefore, bioplastics consisting of natural biopolymers have attracted attention as a method to solve the microplastic problem^21^. Since natural biopolymers possess a biodegradable property, the bioplastics consisting of a biopolymer easily decompose in nature. Additionally, the bioplastic is considered to be extremely non-hazardous to humans and an environmentally-benign material. Furthermore, since the biopolymers can be easily obtained from a natural environment, they are considered to be a sustainable resource. Therefore, the bioplastics consisting of biopolymers, such as starch^22^, casein^23^, lignin^24^, collagen^25^, and keratin^26^, have been reported. Recently, we also reported a water-insoluble and thermal stable bioplastic consisting of soy protein (soy-plastic)^27^. This soy-plastic showed a bending strength value was the same as that of polyethylene (PE). In addition, the soy-plastic showed a biodegradable activity when the soy-plastic was incubated in a proteolytic enzyme-containing aqueous solution. Until now, although the bioplastics consisting of protein and polysaccharide have been reported, those consisting of nucleic acids, such as DNA and RNA, have not been reported to the best of our knowledge.

In this study, we prepared the bioplastics consisting of double-stranded DNA (DNA plastic) and demonstrated their chemical, physical, and biodegradable properties. The DNA plastics were prepared by the immersion of a DNA pellet into a formaldehyde (HCHO) solution and heating. The DNA plastics formed a three-dimensional network by cross-linking between the DNA chains via HCHO and showed a water- and a thermal-stability. The tensile strength of the DNA plastics was the same as that of PE. Additionally, since DNA in plastic possesses the double-stranded structure, these plastics effectively accumulated the DNA intercalator. Furthermore, the DNA plastics indicated a biodegradable property in a nuclease-containing aqueous solution, and the biodegradable stability of the DNA plastic could be controlled by the HCHO concentration.

## Experimental sections

### Material

Double-stranded DNA (sodium salt from salmon milt, molecular weight; > 5 × 10^6^) was obtained from Biochem, Ltd., Saitama, Japan. The formaldehyde (HCHO) solution (37%), ethidium bromide, sodium chloride, calcium chloride dihydrate, and tris-(hydroxymethyl)aminomethane (Tris) were purchased from Wako Pure Chemical Industries, Ltd., Osaka, Japan, or Nacalai Tesque, Inc., Kyoto, Japan. SYBR^®^ Green I and *Micrococcal* nuclease (from *Staphylococcus aureus*) was purchased from Takara Bio, Inc., Shiga, Japan. The plastic film of polyethylene (PE) as a commercial polymer material was obtained from Ube Film, Ltd., Yamaguchi, Japan. Analytical grade solvents were used in all of the experiments. Ultrapure water (Merck KGaA, Darmstadt, Germany) was used in this experiment.

### Quantification of double-stranded DNA content

The quantification of double-stranded DNA content was demonstrated by the following procedure: salmon milt double-stranded DNA was dissolved in 20 mM Tris–HCl buffer (pH 7.4) in the presence of 100 mM NaCl. SYBR^®^ Green I was used as a reagent for detecting double-stranded DNA^28,29^. SYBR^®^ Green I alone does not emit almost fluorescence. However, when SYBR^®^ Green I interacts with double-stranded DNA, these composites show the strong fluorescence^28,29^. The aqueous DNA solution with the addition of SYBR^®^ Green I was analyzed by fluorescence spectroscopy using an F-2500 fluorescence spectrophotometer (Hitachi Co., Ltd., Tokyo, Japan) at 20 °C. The fluorescence spectra were measured at the excitation wavelength of 498 nm. The fluorescence intensity was evaluated at the wavelength of 522 nm. The pBR322 digested DNA^30^, which is composed entirely of double strands, was used as reference DNA. Since the fluorescence intensity is proportional to the content of double-stranded DNA, the content can be calculated from the fluorescence intensity^28,29^. As a result, the content of double strand in salmon milt DNA, that we used, was 78.6%.

### Preparation of bioplastic

The double-stranded DNA (50 mg) was compacted into pellets with a diameter of 13 mm using a hand press (Riken Kiki Co., Ltd., Tokyo, Japan) at a pressure of approximately 600 MPa for 15 min. These pellets were immersed in 0–30% HCHO solutions for 24 h at room temperature. The 0–30% HCHO solutions were prepared by diluting the HCHO aqueous solution with ethanol. These immersed pellets were removed from the HCHO solutions, rinsed with ethanol, dried at room temperature for 24 h, and then heated for 1 h at 80 °C. These processes were repeated twice.

### Swelling ratio of the DNA plastic

The swelling ratio (%) of the DNA plastic was determined by the following procedure: the dried DNA plastics were immersed in water–ethanol mixed solvents for 5 min, then the weight of these swelling materials was measured. The swelling ratio of the DNA plastic was estimated by Eq. (1).

1 $${\text{Swelling ratio }}\left( {\text{\%}} \right) = \frac{{(W_{{\text{s}}} - W_{0} )}}{{W_{0} }} \times 100$$

where *W*_0_ and *W*_s_ are the initial and swelling weights of the DNA plastic, respectively. Since the *W*_s_ – *W*_0_ is 0 when the DNA plastic does not show the swelling, the swelling ratio of Eq. (1) becomes 0. The concentrations of ethanol in the water–ethanol mixed solvent were 0–100% (*v*/*v*). The values of the swelling ratio was expressed as an average of five measurements.

### Tensile strength of DNA plastic

The DNA plastic was cut into 5 × 10 mm^2^ pieces. The thickness of the DNA plastic was measured by an ID-C X series thickness gauge (Mitutoyo Corporation, Kanagawa, Japan). The thickness of the DNA plastic was approximately 0.5 mm. The tensile stress and strain of the DNA plastic was measured using a ZTA-50 N digital force gauge (Imada Co., Ltd., Aichi, Japan) and test stand MX2-500 N (Imada Co., Ltd.). The temperature and relative humidity (RH) during the tensile strength measurements were 20 °C and 50 ± 10%, respectively. The initial length of the DNA plastic was 5 mm and the drawing speed was 10 mm min^−1^. The tensile stress and strain values were expressed as an average of five measurements.

### Structural analysis and staining of DNA plastic

The infrared (IR) absorption spectra of the DNA plastic were characterized using an FT-IR 8400 Fourier transform infrared spectrometer (Shimadzu Corp., Kyoto, Japan) and an IR spectrophotometer FT/IR-4700 (JASCO Corporation, Tokyo, Japan). The IR samples were prepared as follows: the surface of the DNA plastic was scraped to obtain some DNA plastic powder. The obtained powder was mixed with KBr and pelleted by a hand press. The IR spectrum was measured at a resolution of 4 cm^−1^.

The staining of the DNA plastic using SYBR^®^ green I and ethidium bromide was performed by the following procedure: the DNA plastic was immersed in an aqueous SYBR^®^ green I solution (1 drop / 10 ml) or an aqueous ethidium bromide solution (50 µg/ml) for 30 min. This DNA plastic was rinsed with water to remove the non-binding SYBR^®^ green I and non-binding ethidium bromide, then soaked in water. The photography was done during the UV irradiation at 302 nm (UV Transilluminator, TM-10E, Analytik Jena US LLC, Upland, CA).

### Thermal analysis of DNA plastic

The thermal properties of the DNA plastics were analyzed using a DTG-60 thermogravimetric (TG)–differential thermal analyzer (DTA) (Shimadzu Corp.). The TG–DTA samples were prepared as follows: the surface of the DNA plastic was scraped to obtain some DNA plastic powder. The TG–DTA measurement of the DNA plastic powder was carried out at the heating rate of 10 °C min^−1^ from room temperature to 300 °C in flowing dry nitrogen. The sample weights of the TG–DTA measurements were normalized at 1 mg.

### Biodegradable property of DNA plastic

The biodegradable property of the DNA plastic was estimated by the following method^31,32^: the DNA plastic was added to 10 ml of 10 mM Tris–HCl buffer containing 5 mM NaCl and 2.5 mM CaCl_2_ (pH 7.4) in the presence of *Micrococcal* nuclease at 37 °C. The concentrations of the *Micrococcal* nuclease in an aqueous solution were 4–40 units ml^−1^. These DNA plastic-containing samples were incubated for various times at 37 °C. The biodegradable amounts of the DNA plastics were calculated by the absorbance at 260 nm in the absence and presence of the nuclease^31,32^. The biodegradable amounts of the DNA plastics were expressed as an average of three measurements.

## Results and discussion

### Preparation of gellan gum-GPTMS hybrid film

**Figure 1:**
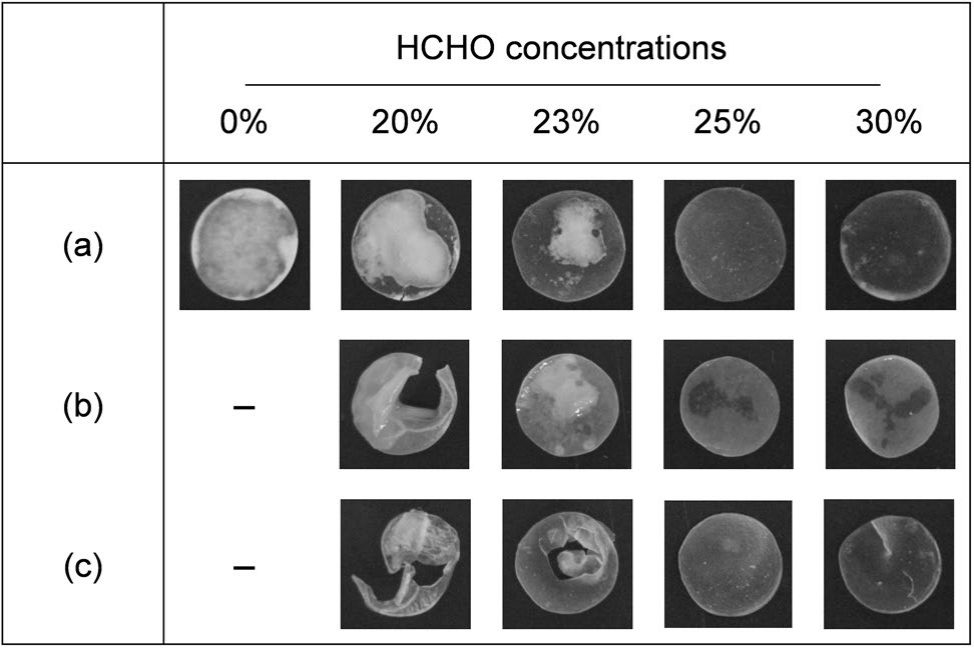
Photograph of DNA plastics which were prepared by various concentrations of HCHO solution. (**a**) DNA plastics which were prepared by the immersion in the HCHO solution, (**b**) DNA plastics which were incubated in water for 24 h at room temperature, and (**c**) dried DNA plastics after immersion in water for 24 h. The symbol – indicates the dissolution of the DNA plastic.

The DNA plastic was prepared by immersing a DNA pellet in 0–30% HCHO solutions for 24 h and heating at 80 °C for 1 h. These processes were repeated twice. The 0–30% HCHO solutions were prepared by diluting an aqueous HCHO solution with ethanol. Figure 1a shows photographs of the DNA plastics which were prepared at the various HCHO concentrations. Additionally, Fig. 1b,c show photographs of the DNA plastics which were incubated in water for 24 h at room temperature and the dried DNA plastic after immersion in water, respectively. When the DNA plastics, which were prepared by the HCHO concentration at < 20%, were incubated in water for 24 h, these plastics completely dissolved in water and disappeared (see Fig. 1b,c). Therefore, these DNA plastics could not be a photographed. Although the DNA plastics, which were prepared at the HCHO concentrations of 20% and 23%, showed no change in formation after several hours of immersion in water, these were partly dissolved in water by immersion for 24 h and the dried DNA plastics showed a significant deformation. When the DNA plastics, which were prepared using 25% and 30% HCHO solutions were incubated in water for 24 h, these plastic showed no evidence of dissolution. Additionally, the dried plastic after immersion in water did not show any deformation. Therefore, we measured the weight loss of the DNA plastic with the 25% HCHO treatment when immersed in water for 24 h at room temperature. As a result, although the DNA plastic was immersed in water for 24 h, the weight loss of the DNA plastic was < 7% and the DNA plastic showed a low solubility. These results suggested that the DNA pellet was water stabilized by the treatment with the HCHO solution. The DNA plastics with the HCHO treatment were stored in ultra-pure water for more than one day to remove the small amount of water-soluble components and then used in further experiments.

On the other hand, we demonstrated the preparation of DNA plastic without the heating treatment. As a result, the DNA plastic without the heating treatment did not show the water-stability and dissolved in water. Similar phenomena have also been reported for bioplastic consisting of soy protein^27^. In this research, the soy protein without the heating treatment did not show the formation of methylene cross-linking. Since similar phenomena, such as the promotion of reaction, occur in DNA plastic, it is necessary to prepare DNA plastic by heating treatment. Additionally, since the salmon sperm double-stranded DNA, that we used, is a natural product, not an artificial synthetic product, not all DNA possesses the double-stranded structure. According to the quantification of double-stranded DNA content using SYBR^®^ green I, the content of double strand in salmon milt DNA, that we used, was 78.6%. These results suggested that the structure of salmon milt DNA is not only double-stranded but also partially collapsed. Therefore, the part of DNA forms the structure which exposed nucleobase. As a result, the distance between nucleobases in the dried condition can be close and the nucleobases can form the cross-linking each other.

### Swelling ratio of DNA plastic

**Figure 2:**
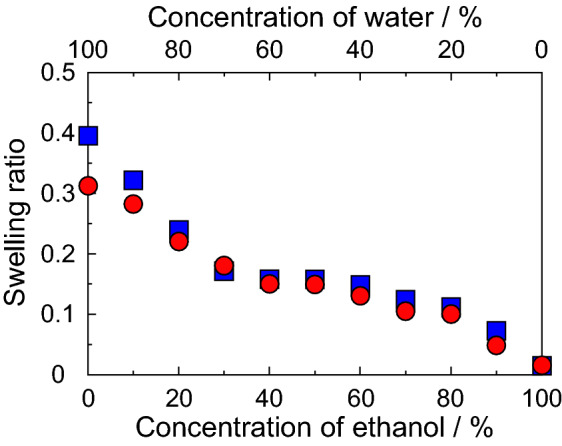
Swelling ratio of (filled square) DNA plastics with 20% HCHO treatment and (filled circle) DNA plastic with 30% HCHO treatment in water–ethanol mixed solvents. The swelling ratio was estimated by Eq. (1). The swelling ratio was expressed as an average of five measurements.

Although the dried DNA plastic after immersion cracked with a slight force and did not show any flexibility, the DNA plastic in water showed more flexibility than the dried DNA plastic. In addition, these DNA plastics underwent swelling when immersed in water. Therefore, we measured the swelling ratio of the DNA plastic in the water–ethanol mixed solutions. The measurements of the swelling ratio of DNA plastics were demonstrated in the water–ethanol mixed solutions. The concentrations of ethanol in the mixed solutions were 0–100%. Figure 2 shows the swelling ratio of (filled square) DNA plastic with 20% HCHO treatment and (filled circle) DNA plastic with 30% HCHO treatment in the water–ethanol mixed solvents. The swelling ratio was estimated by Eq. (1). The swelling ratio of the DNA plastic with 20% HCHO treatment increased with the decrease in the ethanol concentration and indicated the maximum swelling ratio at 0% ethanol (100% water). The value of the swelling ratio in 100% water was approximately 0.4. Similar phenomena were also obtained for the DNA plastic with a 30% HCHO treatment and indicated the maximum value in 100% water. The swelling ratio in 100% water was approximately 0.3 and this value is lower than that of the DNA plastic with a 20% HCHO treatment. These phenomena, such as the increase in the swelling ratio by the increase in the water components, are due to the high water solubility of the DNA. Similar results have been reported for a DNA-inorganic hybrid material using the silane coupling reagents, bis(trimethoxysilylpropyl)amine or bis[(3-trimethoxysilyl)propyl]ethylenediamine^29^, and the swellings of these materials were due to the formation of a dense three-dimensional structure with the cross-linking between the DNA chains. These results suggested that although the DNA plastic slightly swells under the water condition, the swelling ratio decreased with the increase of the HCHO concentration. In addition, the decrease in the swelling ratio due to the increase in the HCHO concentration indicated the formation of a dense three-dimensional structure in the DNA plastic. On the other hand, since the DNA plastic without the HCHO treatment is soluble in water, the swelling ratio at 100% water was not able to be measured.

### Tensile strength of DNA plastic

**Figure 3:**
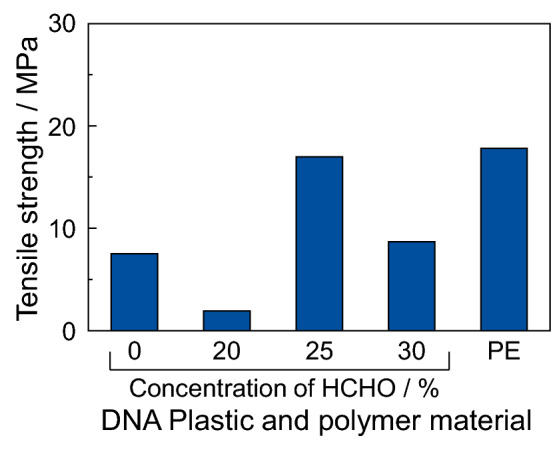
Tensile strength of DNA plastics with the treatment of various HCHO concentrations and polyethylene (PE) material. The initial length of the DNA plastic and PE material was 5 mm and the drawing speed was 10 mm min^−1^. The tensile strength was expressed as an average of five measurements.

The DNA plastics were prepared by immersion in the HCHO solution and the water stability of the DNA plastics increased with the HCHO treatment concentration. Therefore, we measured a physical property, such as the tensile strength, of the DNA plastic. The initial length of the DNA plastic was 5 mm and the drawing speed was done at 10 mm min^−1^. The water-soluble DNA plastic without the HCHO solution treatment has been used as a control of the tensile strength measurement. When the non-treated DNA plastic was loaded at the stress of approximately 7.5 MPa, this material broke. Therefore, this stress at the break was defined as the ultimate tensile strength of the DNA plastic. Figure 3 shows the ultimate tensile strength of the DNA plastics, which were prepared using various HCHO concentrations, and of the polyethylene (PE) material as a reference polymer material. The ultimate tensile strength of the DNA plastic with the 20% HCHO treatment was lower than that of the non-treated DNA plastic. This is due to as follows: it is known that the double-stranded DNA structure changes from the B-form to the A-form in high ethanol concentration or dried condition and the length of DNA becomes shorter by compressing the structure^1,33^. Therefore, the molecular length of DNA in DNA plastic with 20% HCHO treatment is shorter than that of non-treated DNA. As a result, the tensile strength of the DNA plastic with 20% HCHO treatment decreased. The DNA plastics with 25% and 30% HCHO treatments showed a higher tensile strength than the non-treated DNA plastic. Especially, the tensile strength of the DNA plastic with the 25% HCHO treatment was approximately 17 MPa and this value was 2.2 times higher than that of the non-treated DNA plastic. In addition, the tensile strength of the DNA plastic with the 25% HCHO treatment was the same as that of the PE material. In contrast, the tensile strength of DNA plastic at 30% HCHO treatment was lower than that of 25% HCHO treatment. This is due to as follows: at 30% HCHO treatment, the HCHO molecules reacted on the surface of a DNA pellet and the HCHO molecules could not penetrate into the pelleted material. As a result, the cross-linking was produced only on the surface of DNA pellet and the tensile strength of DNA plastic at 30% HCHO treatment become lower than that of 25% HCHO treatment. These phenomena have been reported a bioplastic consisting of soy protein^27^. These results suggested that the DNA plastic with the 25% HCHO treatment shows a high physical strength.

On the other hand, the elongation at the break point of the DNA plastic with the HCHO treatment was approximately 10%. The elongation of the DNA plastic with 25% and 30% HCHO treatments was both approximately 15% and these values were almost the same as that of the non-treated DNA. These results suggested that the DNA plastic does not possess a flexibility under the dry condition. Furthermore, we calculated the cross-linking density of DNA plastic (see Section 1.1 in the Supplementary Information). As a result, the DNA plastic indicate the high cross-lining density.

### Molecular structure of DNA plastic

**Figure 4:**
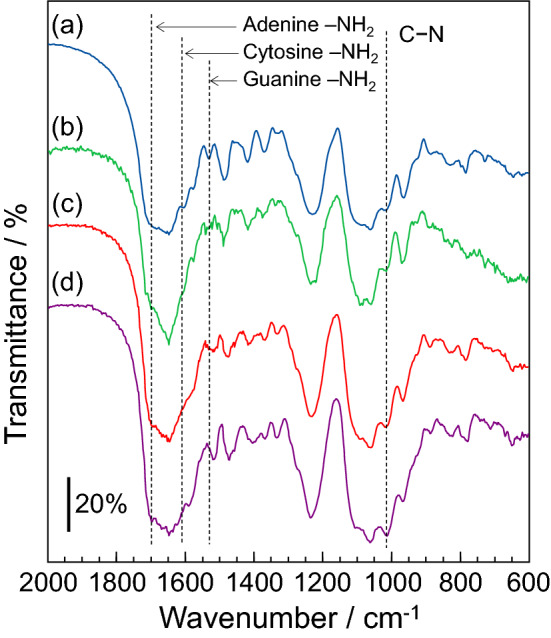
IR spectra of DNA plastics which were prepared by the immersion in HCHO solutions of (**a**) 0% (non-treated), (**b**) 10%, (**c**) 20%, and (**d**) 30%. The IR spectrum was measured at the resolution of 4 cm^−1^. The scale bar indicates the transmittance of 20%. Triplicate experiments gave similar results.

The water-insoluble DNA plastics were prepared by the immersion in the HCHO solution. The molecular structure of the bioplastic consisting of DNA was confirmed by IR spectrometry using the KBr method. The IR sample was prepared by scraping the surface of the DNA plastic. Figure 4 shows the IR spectra of the DNA plastics which were prepared by immersion in the HCHO solutions of (a) 0% (non-treated), (b) 10%, (c) 20%, and (d) 30%. The DNA plastics with the HCHO treatment showed an absorption band at ca. 1000 cm^−1^ related to the stretching vibration of C–N^34,35^. The intensity of the absorption band at ca. 1000 cm^−1^ increased with the HCHO concentration (see Section 1.2 and Figure S1 in Supplementary Information). In contrast, the non-treated DNA indicated absorption bands at 1603 cm^−1^ and 1529 cm^−1^, attributed to the scissoring vibration of –NH_2_ in cytosine and guanine, respectively^36–38^. In addition, the non-treated DNA showed an absorption band at ca 1690 cm^−1^ due to the scissoring vibration of –NH_2_ in adenine^38,39^. These absorption bands at 1690 cm^−1^, 1603 cm^−1^, and 1529 cm^−1^ decreased or disappeared with the increase in the HCHO concentration (see Section 1.2 and Figure S1 in Supplementary Information). These results suggested that the amino groups of the nucleobase in the DNA form the C–N bonding by the reaction with HCHO. Generally, in the biopolymer, the HCHO molecules react with the amino group and produce the methylol derivative of the amino group. These methylol derivatives react with other amino groups in the biopolymer and form the methylene cross-linking, such as N–CH_2_–N^40–42^. Therefore, the methylene-crosslinking occurred between the double-stranded DNA chains and formed the DNA plastic with a three-dimensional network. These methylene cross-linkings with the formation of the three-dimensional network provided DNA the water-stability and the mechanical strength. Similar phenomenon, such as the formation of the C–N bonding by the reaction with the HCHO, has been reported for a bioplastic consisting of soy protein^27^ and an accumulation of HCHO molecules by the nucleic acid^43^. Furthermore, the DNA plastic indicated high cross-linking density (see Section 1.1 in the Supplementary Information). These results suggested that not only the methylene cross-linking but also the hydrogen bonding in DNA is attributed to the formation of DNA plastic.

On the other hand, the non-treated DNA material indicated an absorption band at 1229 cm^-1^ related to the phosphate group of A-fomed DNA with a double-stranded structure^44,45^ (see spectrum (a) in Fig. 4). This absorption band did not change despite the HCHO treatment. Generally, the absorption band related to the phosphate group significantly varies when a conformational change in the DNA occurs^6,44,46^. Additionally, the DNA plastic showed the absorption band at 900 cm^−1^, attributed to the deoxyribose ring vibration of A-formed DNA. These results suggested that the DNA in the dried DNA plastic is predominantly A-form and possesses the double-stranded structure.

**Figure 5:**
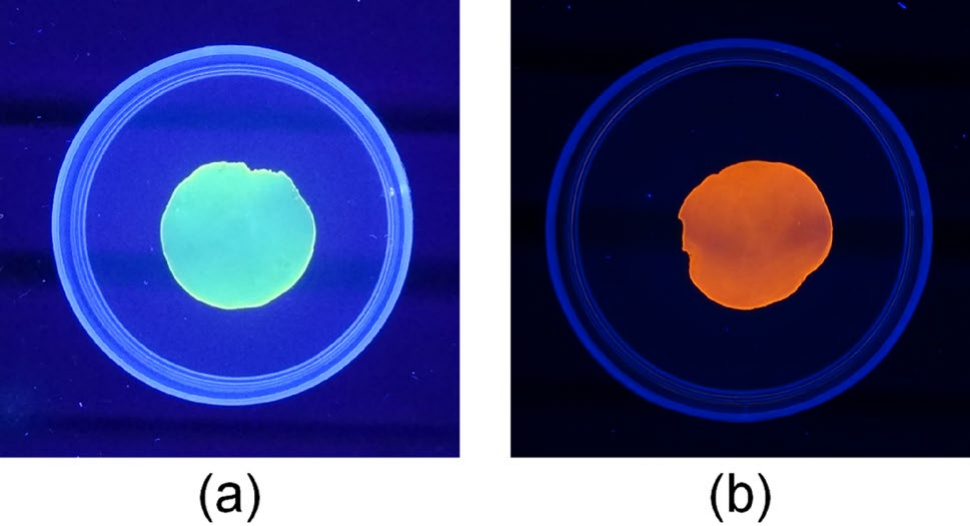
Fluorescence images of DNA plastic with staining by (**a**) SYBR^®^ green I and (**b**) ethidium bromide during 302 nm UV irradiation. The DNA plastic was a sample with a 25% HCHO treatment.

Generally, the ethanol solution induces the denaturation of DNA and the DNA structure changes from B-formed DNA, which is native DNA structure in an aqueous solution, to A-formed DNA, which is formed under dehydrated condition^1^. This A-formed DNA changes to B-formed DNA again under aqueous solution or high humidity conditions^1^. In contrast, SYBR^®^ green I, which alone does not emit the fluorescence, interacts specifically with double-stranded DNA and indicates a strong fluorescence during the UV irradiation^28,29^. The ethidium bromide, which is one of the famous DNA intercalators, intercalates in the B-formed DNA and indicates a strong fluorescence during the UV irradiation^1^. Therefore, we estimated the DNA structure in DNA plastic by the interaction of SYBR^®^ green I and ethidium bromide. Figure 5a,b show the fluorescence images of the SYBR^®^ green I- and the ethidium bromide-stained DNA plastic by the 25% HCHO treatment during the UV irradiation at 302 nm, respectively. The SYBR^®^ green I-stained DNA plastic showed a strong green fluorescence and the DNA plastic possessed a double-stranded structure. The ethidium bromide-stained DNA plastic showed a strong red fluorescence by the intercalation of ethidium bromide into B-formed DNA, which is native DNA structure, during the UV irradiation. As a result, the DNA structure in DNA plastic possesses B-form under aqueous solution and the denaturation by the immersion in HCHO solution diluted with ethanol did not significantly affect the DNA structure in DNA plastic except for the methylene cross-linking between DNA chains. In addition, the strong fluorescence appeared over all the DNA plastic and the non-shading was shown. These results suggested that the cross-linking reaction between the DNA chains rarely affected the double-stranded structure under our reaction conditions, and that the three-dimensional network consisting of the double-stranded DNA was homogeneously formed in the DNA plastic.

### Thermal stability of DNA plastic

**Figure 6:**
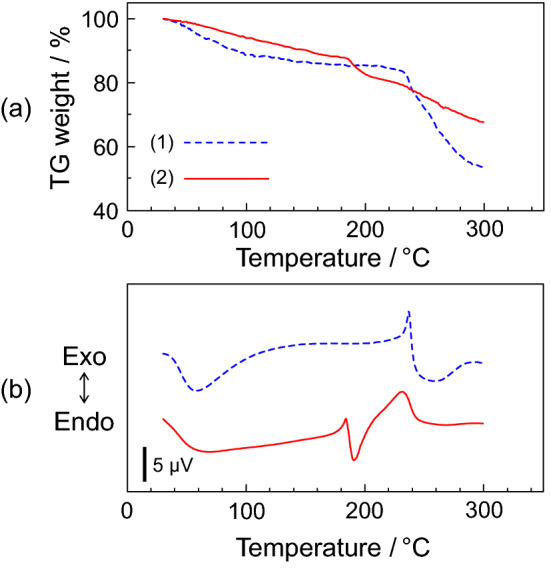
(**a**) TG and (**b**) DTA curves of (1) non-treated DNA and (2) DNA plastic with the 25% HCHO treatment at the heating rate of 10 °C min^−1^ to 300 °C under flowing dry nitrogen. The sample weights of the TG–DTA measurements were normalized at 1 mg. Triplicate experiments gave similar results.

The DNA plastics were formed by the cross-linking reaction between the DNA chains. Therefore, the thermal stability of the DNA plastic was measured by the TG–DTA. Figure 6 shows the (a) TG and (b) DTA curves of (1) the non-treated DNA and (2) the DNA plastic made by the 25% HCHO treatment. The TG–DTA measurements were demonstrated at the heating rate of 10 °C min^−1^ up to 300 °C under flowing dry nitrogen. The non-treated DNA showed the TG weight loss of approximately 10% and a large endothermic peak at < 100 °C. This is due to the evaporation of water and a similar result has already been reported^26^. In addition, at 233.28 °C, the non-treated DNA showed an exothermic peak related to the pyrolysis. In contrast, the DNA plastic with the 25% HCHO treatment showed endothermic peaks attributed to the evaporation of water from plastic at < 100 °C. In addition, an endothermic peak at 184.01 °C appeared. Similar endothermic peaks have also been reported for soy-plastic made by the HCHO treatment^27^. Therefore, these endothermic peaks are due to the decomposition of the substance, which was produced by the reaction with the HCHO molecules. These results suggested that the DNA plastic with the HCHO treatment possessed a thermal stability at < 150 °C.

### Biodegradable property of DNA plastic

DNA plastic with the HCHO treatment showed a water-stability and mechanical stability by cross-linking between the DNA chains. Finally, we demonstrated the biodegradable property of the DNA plastic using *micrococcal* nuclease, which is one of the DNA-hydrolyzing enzymes^1,31,32^. The biodegradation of the DNA plastic was performed at 37 °C. The biodegradable amounts of the DNA plastics by the enzyme reaction were calculated from the absorbance at 260 nm^31,32^.

The concentration of nuclease varies greatly depending on the type of water. However, the concentrations of DNA released into water from freshwater fish and saltwater fish have been reported to decrease by approximately 10% and 5% per hour, respectively^47–49^. Additionally, it has also been reported that the degradation of DNA is faster when the water temperature is high^50^. On the other hand, one unit of nuclease is defined as completely degrading 1 µg of DNA in 1 h. Therefore, in our biodegradable condition of 40 units/ml, 5.3% of DNA plastic is calculated to be degraded in 1 h. It is almost the same as the value of saltwater fish.

**Figure 7:**
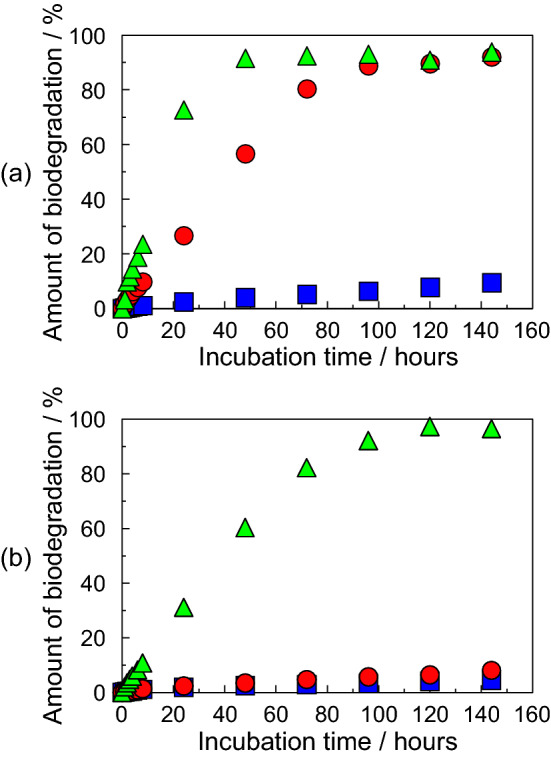
The biodegradations of the DNA plastics which were prepared by the immersion in (**a**) 25% HCHO and (**b**) 30% HCHO solutions. The (filled square), (filled circle), and (filled triangle) denote the enzymatic concentrations of 4 units ml^−1^, 10 units ml^−1^, and 40 units ml^−1^, respectively. The amount of biodegradation was expressed by an average of three measurements.

Figure 7a,b show the biodegradation of the DNA plastics, which were prepared at 25% and 30% HCHO solutions, respectively, in a *micrococcal* nuclease-containing aqueous solution. The (filled square), (filled circle), and (filled triangle) in Fig. 7 indicate the degradation in the enzymatic concentration of 4 units ml^−1^, 10 units ml^−1^, and 40 units ml^−1^, respectively. In the enzyme concentration of 4 units ml^−1^, the amount of biodegradation of the DNA plastic with the 25% HCHO treatment slightly increased with the incubation time and the amount of the biodegradation at 144 h was less than 10% (see filled square in Fig. 7a). Therefore, we demonstrated the biodegradation of the DNA plastic at a higher enzymatic concentration, such as 10 units ml^−1^ or 40 units ml^−1^. As a result, the amount of biodegradation increased with the enzymatic concentration and these amounts at 10 units ml^−1^ and 40 units ml^−1^ reached constant values at 98 h and 48 h, respectively. These biodegradation amounts were more than 90%. As a result, the DNA plastic by the 25% HCHO treatment was able to be almost degraded by controlling the enzymatic concentrations. Similar biodegradable measurements were also demonstrated for the DNA plastic which was prepared by the 30% HCHO solution. The biodegradation amounts at the enzymatic concentrations of 4 units ml^−1^ and 10 units ml^−1^ were less than 10% at the incubation time of 144 h (see filled square and filled circle in Fig. 7b). When the biodegradation of the DNA plastic at the enzymatic concentration of 40 units ml^−1^ occurred, the amount of biodegradation increased with the incubation time and reached a constant value at approximately 120 h. These values were more than 95% and the DNA plastic was almost decomposed. These results suggested that the DNA plastics with the HCHO treatment possess biodegradable properties. Furthermore, the biodegradable stability of the DNA plastic could be controlled by the HCHO concentration.

## Conclusion

We prepared a water-insoluble and thermally stable DNA plastic by immersion of the DNA pellet in an HCHO solution. The DNA plastic showed a high mechanical strength by the formation of a three-dimensional network by the crosslinking reaction between the DNA chains, and its tensile strength was the same as that of PE. In addition, since the DNA plastic was able to accumulate SYBR^®^ green I and ethidium bromide, the DNA in the DNA plastic possessed a double-stranded structure and a function of intercalation. Furthermore, the DNA plastic underwent biodegradation in a nuclease-containing aqueous solution and its biodegradable stability could be controlled by the HCHO concentration. Since the DNA intercalators have been used for medical drugs, antibacterial agents, dyes, photo-functional molecules, etc., the intercalator-containing DNA plastics are expected to have a release effect with the biodegradation. Therefore, the DNA plastic with the biodegradable property may have potential use in environmental, agricultural, biomedical, engineering applications, and outdoor leisure products, such as golf tees and fishing fake baits.

## Supplementary Information

Supplementary Information.
