# Supplementary material for: Preparation of bioplastic consisting of salmon milt DNA
Source: Sci Rep. 2022 May 6;12:7423. doi: 10.1038/s41598-022-11482-4 (PMC9076882; doi:10.1038/s41598-022-11482-4)
Supplement: Supplementary file 1 — Supplementary Information. [file 41598_2022_11482_MOESM1_ESM.docx]

**Supplementary Information**

Preparation of bioplastic consisting of salmon milt DNA

Masanori Yamada^*,1^ and Midori Kawamura^1^, and Tetsuya Yamada^2^

^1^Department of Chemistry, Faculty of Science, Okayama University of Science, Ridaicho, Kita-ku, Okayama 700-0005, Japan

^2^Research Faculty of Agriculture, Hokkaido University, Kita 9, Nishi 9, Kita-ku, Sapporo, Hokkaido, 060-8589, Japan

[*] Corresponding author

Department of Chemistry, Faculty of Science, Okayama University of Science, Ridaicho, Kita-ku, Okayama 700-0005, Japan

Tel: +81 86 256 9550

Fax: +81 86 256 9550

E-mail: myamada@chem.ous.ac.jp

**1.1. Cross-kinking density of DNA plastic**

We evaluated the cross-linking density assuming the DNA plastic is based on affine network^1-4^. The cross-linking density was calculated from following equation.

$$G=\frac{\rho RT}{M_{net}}$$

where *G* is the elastic modulus, *ρ* is the density of material, *R* is the gas constant, *T* is the absolute temperature, and *M*_net_ is the number average molecular weight of network. The measurement of the elastic modulus was demonstrated as following: DNA plastics, which were prepared by various HCHO concentrations, were immersed in pure water and were swelled. The tensile strength of swelled DNA plastic was measured using a force gauge. The elastic moduli of swelled DNA plastic with the 20%, 25%, 30% HCHO treatments were 808 KPa, 14.5 MPa, and 1.83 MPa, respectively. These values of elastic modulus were high since the swelling ratio of DNA plastic was not high. Additionally, the calculated cross-linking densities of DNA plastic with the 20%, 25%, 30% HCHO treatments were 331 mol m^-3^, 5950 mol m^-3^, and 750 mol m^-3^, respectively. Furthermore, we calculated the average distance between cross-linkings from the cross-linking density. As a result, the calculated average distances between cross-linkings of DNA plastic with the 20%, 25%, 30% HCHO treatments were 1.71 × 10^-9^ m, 6.54 × 10^-10^ m, and 1.31 × 10^-9^ m, respectively. These cross-linking densities were high values. In addition, the average distances between cross-linkings were extremely short. These results suggested that not only the methylene cross-linking with the HCHO treatment but also the hydrogen bonding in DNA is attributed to the formation of DNA plastic.

**1.2. Intensity ratio of IR absorption band**

Figure S1 shows the intensity ratio of IR absorption bands at 1690 cm^-1^, 1603 cm^-1^, 1529 cm^-1^, and ca. 1000 cm^-1^ as a function of HCHO concentration. The intensity ratio was estimated from equation (S1).

$$Intensity ratio= \frac{I_{conc.}}{I_{0}}\times100 (S1)$$

where *I*_0_ and *I*_conc._ are intensity of IR absorption bands at non-treated DNA and DNA plastic with HCHO treatment, respectively. As a result, the intensity ratio at 1690 cm^-1^, 1603 cm^-1^, and 1529 cm^-1^, which are related to the scissoring vibration of amino group of nucleobase^5,6^, decreased with the HCHO concentration. The intensity ratio at ca. 1000 cm^-1^, which is related the stretching vibration of C−N, increased with the HCHO concentration.

**Figure S1.** Intensity ratio of IR absorption bands at (●) 1690 cm^-1^, (▲) 1603 cm^-1^, (■) 1529 cm^-1^, and (○) ca. 1000 cm^-1^ as a function of HCHO concentration. Intensity ratios were estimated from equation (S1). Since the absorption bands at 1529 cm^-1^ disappeared in 20% and 30% HCHO treatments, these plots were not shown in Figure S1.

References

1. Sato, T., Kanaya, T., Ito K., Watanabe, H., Tanaka, K., Shimomura, T. & Inoue, T. Structures and Physical Properties of Polymers (in Japanese), (Kodansha Ltd., 2013).

2. Indei, T., Schieber, J. D. & Takimoto, J. Effects of fluctuations of cross-linking points on viscoelastic properties of associating polymer networks, *Rheol. Acta* **51**, 1021-1039 (2012).

3. Topuz, F. & Okay, O. Rheological behavior of responsive DNA hydrogels. *Macromolecules* **41**, 8847-8854 (2008).

4. Nagy, M. Some new aspects of research on polymer gels. *Colloid Polym. Sci.* **263**, 245-265 (1985).

5. Nakamoto, K., Tuboi, M. & Strahan, G. D. Drug-DNA Interactions. Structures and Spectra (John Wiley & Sons, 2008).

6. Hirakawa, A. Y., Okada, H., Sasagawa, S. & Tsuboi, M. Infrared and Raman spectra of adenine and its ^15^N and ^13^C substitution products. *Spectrochim. Acta A* **41**, 209-216 (1985).
